# Nocturnal One-Hour Lighting Stimulates Gonadal Development and Lowers Fat Deposition in Male Mule Ducks

**DOI:** 10.3390/ani11030614

**Published:** 2021-02-26

**Authors:** Tz-Chuen Ju, Kai-Chien Tsao, Tzu-Yu Liu, Shyi-Kuen Yang

**Affiliations:** Department of Animal Science and Biotechnology, Tunghai University, Taichung 40704, Taiwan; tzchuen@thu.edu.tw (T.-C.J.); S06610224@thu.edu.tw (K.-C.T.); jane890325@gmail.com (T.-Y.L.)

**Keywords:** fat deposition, gonadal development, mule duck, photoperiod

## Abstract

**Simple Summary:**

Photoperiods can affect sexual maturity, body weight, and body composition. In this work, we provided male mule ducks with a one-hour lighting from 20:00 to 21:00, in addition to the natural photoperiod, and evaluated its effects on their body weight, organ mass, gonadal function, and plasma levels of metabolites. The results indicate that the nocturnal lighting stimulated gonadal development and function and reduced fat deposition. This implies that nocturnal lighting is able to shorten the feeding period for the marketing of mature ducks.

**Abstract:**

In this study, the effects of a nocturnal light pulse on body weight, organ mass, gonadal function, and plasma levels of metabolites were determined in male mule ducks. In total, 32 15-week-old mule ducks were randomly allocated to either Group C (control group) or L+ (lighting group). Group C was exposed to the natural photoperiod, whereas Group L+ was provided with a 1-h lighting over 20:00–21:00 every day, in addition to the natural photoperiod. At the end of the 42-day experiment, Group L+ had significantly lower relative weights (% of live weight) of the digestive tract and abdominal fat and higher relative weights of the breast meat and testes than Group C. Moreover, Group L+ had significantly higher plasma testosterone and lower plasma glucose levels. However, no between-group differences were observed in the triacylglycerol and uric acid levels. Histological examination demonstrated that the seminiferous tubule diameter was larger in Group L+ than in Group C. Moreover, the meiosis stage in spermatogenesis had begun in Group L+ but not in Group C. In conclusion, the supplemented 1-h lighting at 20:00 stimulated gonadal development and function and reduced fat deposition.

## 1. Introduction

The mule duck (or mulard) is a sterile hybrid between a Muscovy drake (*Cairina moschata*) and a mallard duck (*Anas platyrhynchos*). Muscovy ducks are large ducks, native to Mexico and Central and South American countries, which breed in the spring and summer. Domestic ducks are believed to be derived from wild mallard ducks. Wild mallard ducks are migratory birds, which breed in the northern breeding range and winter in the south. Before migration, the feed intake and fat deposition increase in many bird species [1]. Most birds, including migratory birds, demonstrate a seasonal reproductive pattern for the maximum survival rates of their offspring. The annual cycle of the photoperiod is the most crucial zeitgeber of the circannual rhythms, such as fat deposition, molt, reproduction, and migration, in almost all bird species living at high altitudes [2,3,4], even the temperate zone species which are exposed to near-equatorial photoperiods [5] and the subtropical species which are exposed to programmed photoperiodic schedules [6]. 

Domestication may have reduced but not repressed the seasonality of these birds. For instance, the captive undomesticated Muscovy ducks lay from February to September in England (in the Northern Hemisphere) [7], and the domesticated Muscovy ducks typically lay from August to April or May in Mozambique (in the Southern Hemisphere) [8]. The gonadal recrudescence and maturation of Muscovy ducks are controlled by the photoperiod [9,10]. In Pekin ducks, which were domesticated from the wild mallard, the seasonality of reproduction is retained, and their gonadal recrudescence is photosensitive to the increasing day length [11]. The photoperiod also controls changes in body weight, fat deposition, and feed intake. In general, shorter days promote fat deposition and longer days stimulate protein accretion in domestic animals [12].

Mule ducks are the most common species of duck consumed for meat in Taiwan. They grow rapidly, attaining near-mature body weight early in life, and thus are sold at 10 weeks of age. However, they may also be sold at >14 weeks of age as replacement for mature male Muscovy ducks, which are used for cooking ginger duck stew, a main course consumed in winters. Considering their rapid growth early in life, understanding the effects of the photoperiod on fat deposition in mule ducks may be more helpful than understanding those on weight gain or protein accretion after the conventional fattening period. In this study, we investigated the effects of supplemented light in the scotophase (i.e., skeleton long photoperiod) during short days on the body weight, body composition, and testis development in male mule ducks. Plasma levels of metabolites associated with nutrient metabolism were also measured.

## 2. Materials and Methods

### 2.1. Animals

The experimental animals used in this study were three-way crossbred male mule ducks (Muscovy × [Pekin × Tsaiya]), which were originally purchased from a commercial hatchery. They were grown on an elevated plastic mesh floor without litter and exposed to a natural photoperiod in another barn of the experimental farm of Tunghai University before the experiment. They were transferred to the experimental barn and assigned to the experiment when they were 105 days old. 

### 2.2. Estimation of Sample Size

Based on the deduction from the independent two-sample t-test, the sample size per group (n) for a 5% level of significance was calculated by the formula below:

n = 2 × (t_0.05_)^2^ × (SD/ES)^2^

where t_0.05 (14)_ = 1.76; t_0.05 (30)_ = 1.70.

SD is the predicted standard deviation; ES is the predetermined effect size. Since a previous study showed that the standard deviation of testicular weights in ducks, which is the most important parameter in this study, was small (ranged from o.1 to 3.1 g) [13], and we anticipated that the photoperiodic effect would be large, ES was anticipated to be equal to or larger than SD. Therefore, 7 ducks per group should be enough for the comparison in testicular weight. The photoperiodic effect on the weight of abdominal fat, an important parameter in this study, was also anticipated to be large, and 7 ducks per group should be enough for the comparison between groups. Therefore, 8 ducks per group were killed for the collection of related data. However, the SD of body weight ranged from 222 to 232 g [14], and we anticipated that the photoperiodic effect would be moderate; therefore, ES was anticipated to be 60% of SD. Therefore, 16 ducks per group were required.

### 2.3. Experimental Design

We allocated 32 male mule ducks to either Group C (control group) or Group L+ (lighting group) on 3 October. Each group was kept in a separated room comprising four 125 × 180 cm pens, with each pen containing four ducks. Group C was exposed to the natural photoperiod, which was decreasing in day length, whereas Group L+ was provided an artificial lighting using fluorescent tubes over 20:00–21:00 every day, in addition to the natural photoperiod. The intensity of the light on the floor during the lighting period ranged from 55 to 60 lux. During the experimental period (from 3 October to 13 November), the length of the day (civil dawn to civil dusk) of the natural photoperiod decreased from 12.6 to 11.8 h, and the daily mean ambient temperature fluctuated between 21.1 and 28.6 °C.

### 2.4. Management and Data Collection

During the experimental period, the ducks were raised on concrete floors without litter, and the floor was cleaned once daily. They were fed ad libitum on a fattening diet (Fwusow, Taichung, Taiwan), mainly composed of corn meal and soybean meal and containing >14% CP, >3% fat, <7.0% crude fiber, <13% moisture, 0.75% calcium, and 0.65% phosphorus. Feed intake per pen was recorded daily, and body weight was recorded weekly. Neck circumference (at the thinnest area) was also recorded weekly from 126 days of age. Before weighing, the ducks were deprived of the feed for 12 h. At the end of the experiment (147 days of age), ducks were fasted for >8 h followed by blood sampling from the metatarsal vein by venipuncture for the determination of plasma levels of glucose, uric acid, triacylglycerol, and testosterone. To minimize the stress, the ducks were sequentially transferred to a plastic bucket with a cover when they were weighed and were covered with black cloth on the head when their blood samples were collected.

### 2.5. Sample Analyses

The plasma levels of metabolites were determined on an automatic blood analyzer (VITROS DT60 II Chemistry System; *Johnson & Johnson* Ortho *Clinical* Diagnostics). Plasma testosterone levels were determined on a commercial enzyme-linked immunosorbent assay kit (Cayman, Ann Arbor, MI, USA). The assay sensitivity was 6 pg/mL, and the intra- and inter-assay CVs were 4.4% and 7.7%, respectively. After blood sampling, two ducks with a body weight close to the average body weight in each pen were sacrificed through exsanguination to measure the organ weight, analyze the chemical composition of the breast meat, and histologically examine the testes. The other two were reserved for another experiment. The chemical compositions of the breast meat were determined using the procedures of the Association of Official Chemists (AOAC, 2003). The testis samples were fixed in 4% paraformaldehyde solution, dehydrated in ethanol, and embedded in paraffin blocks, which were then cut into 5-µm-thick sections and stained with hematoxylin and eosin.

### 2.6. Statistical Analysis

In the statistical analysis, each duck was considered an experimental unit, except in the analysis of feed intake data, where each pen was considered an experimental unit. The mean values were compared between groups using Student’s *t* test. The criterion of statistical significance was set at *p* < 0.05.

## 3. Results

The body weights in both groups decreased during the experimental period, and the difference in the final body weight between groups was not significant (Table 1). The body weights of the mule ducks decreased during the first week of the experiment, especially in Group L+ (data not shown). No difference in weight loss during the experimental period between groups was found (Table 1). The daily feed intake fluctuated during the experimental period (data not shown), but there was no between-group difference in the average feed intake of the entire experimental period (Table 1). In the final week, there was no between-group difference in the neck circumference (Table 1).

When the ducks were killed at 147 days of age, Group L+ demonstrated higher organ weight and relative weight (100% × [organ weight/live body weight]) of the testes, but lower organ and relative weights of abdominal fat and the empty digestive tract than Group C. There were no between-group differences in the weights of the breast meat, liver, and heart; however, the relative weights of the breast meat and heart were higher in Group L+ than in Group C (Table 2). Nevertheless, there was no between-group difference in the breast meat composition (Table 3). Fasting plasma glucose levels were lower in Group L+ than in Group C (Table 4). However, fasting plasma triacylglycerol and uric acid levels were not affected by the photoperiod. Plasma testosterone levels were higher in Group L+ than in Group C (Table 4).

**Figure 1:**
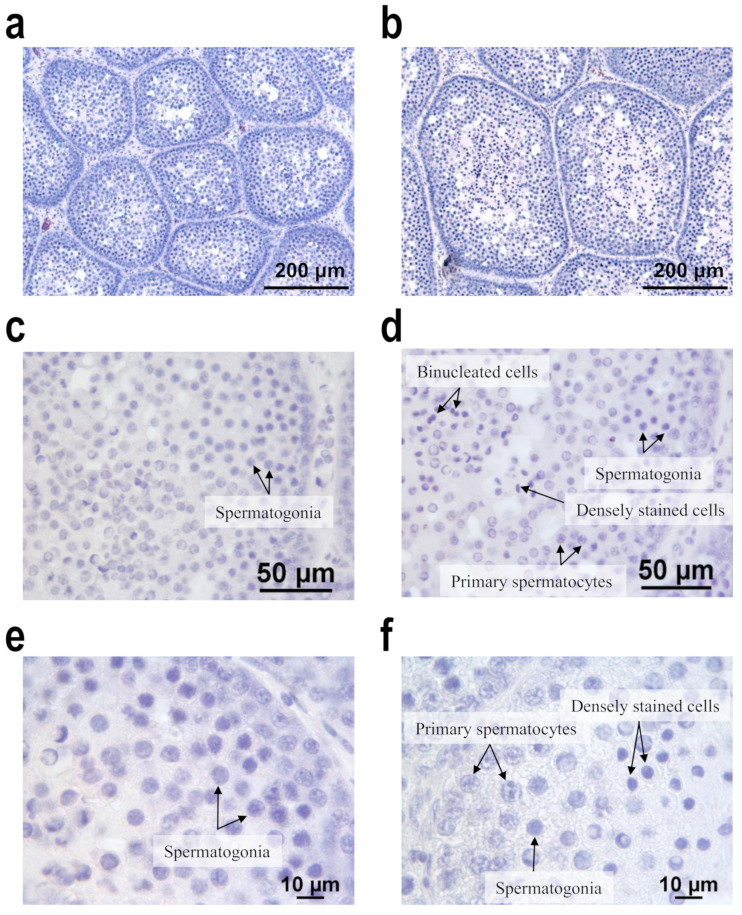
Effects of the nocturnal 1-h lighting on the histological characteristics of testes of our mule ducks. (**a**) Section from the testis of a mule duck in Group C displays thin seminiferous tubules. (**b**) Section from Group L+ reveals thick seminiferous tubules. (**c**,**e**) Section from Group C does not exhibit spermatogenesis, and only spermatogonia exist. (**d**,**f**) Section from Group L+ displays active spermatogenesis, and primary spermatocytes, as well as spermatogonia, are present. In addition, some small deeply stained cells and binucleated cells are present in the central area of the tubule in sections from Group B. No spermatozoa are observed in any sections. Group C was subjected to the natural photoperiod, and Group L+ was supplemented with 1-h artificial light from 20:00 to 21:00, in addition to the natural photoperiod. Hematoxylin and eosin stain.

Histological examination demonstrated that the seminiferous tubule diameter was larger in Group L+ than in Group C (Figure 1a,b). Spermatogenesis had commenced in Group L+, with all samples displaying several primary spermatocytes as well as spermatogonia (Figure 1d). Some small cells with small intensely stained nuclei and multinucleated cells were observed in the central areas of the tubules, and lumen had started developing (Figure 1f). However, Group C demonstrated only spermatogonia because spermatogenesis had not commenced (Figure 1c,e). No spermatozoa were observed in either group (Figure 1). Moreover, at 126 days of age, 9 of the 16 ducks in Group L+ exhibited active mounting behavior, but no Group C duck exhibited this behavior (data not shown). At 119 days of age, the bills of 14 of the 16 ducks in Group L+ had started to change color from pale pink to red at the caudal border, whereas the bills of only 7 of the 16 ducks in Group C had begun turning orange. By the final week, the color of the bills in both groups was changing to red or orange; however, empirically, the bills had more area becoming red in Group L+ than in Group C.

## 4. Discussion

The growth curve of a mule duck is sigmoid in shape, with an inflection point between 25 and 30.5 days for male mule ducks [15,16]. Their body weights peak at 12 weeks of age and decrease thereafter [16]. Therefore, at the beginning of this experiment, the body weights of ducks had attained somatic maturity. In this study, mule ducks in both groups lost their body mass during the first week of the experiment. The weight loss may be attributed to the stress caused by the change in environment or experimental operation. The final body weights in both groups did not recover to the initial weights. In this study, the body mass was not significantly affected by the photoperiod regimen. 

In this study, Group L+ had higher relative heart weights than Group C, even though there was no difference in the absolute weights between groups. The higher relative heart weight was consistent with the higher breast meat weight (Table 2). In sparrows, the breast meat mass is positively correlated with the heart mass [17]. The heart mass may reflect the metabolic demand. In general, skeletal muscles are more metabolically active than other tissues, particularly the adipose tissue. Here, Group L+ demonstrated lighter digestive tracts than Group C. Since their daily feed intake did not differ from that of Group C and their relative digestive tract weight was lower than that of Group C, the food passage rate in the digestive tract in Group L+ was speculated to be higher than that in Group C. Therefore, our results demonstrate that a nocturnal 1-h lighting resulted in high relative weights of the breast meat and heart and a low relative weight of the digestive tract and implied that the photoperiodic regimen increased the food passage rate.

In this study, the precocious testis development in Group L+ was accompanied by active mounting behavior and high plasma testosterone levels. High plasma testosterone levels might contribute to the active mounting behavior. Higher testosterone levels might result in higher relative breast meat weight and lower fat deposition in Group L+ compared with Group C. In general, testosterone promotes aggressive and sexual behaviors, protein accretion, and decreases in fat deposition. In domestic animals such as cattle, sheep, and pigs, castration increases the ratio of fat mass-to-lean mass [18]. In chickens, caponization enhances hepatic lipogenesis, whereas testosterone implantation reduces hepatic lipogenesis and lipid accumulation [19,20]. An in vitro study reported that androgens stimulate myogenic differentiation and inhibit adipogenesis in mesenchymal pluripotent cells via an androgen receptor-mediated pathway [21]. Here, the nocturnal 1-h lighting possibly caused a decrease in the relative weight of abdominal fat and an increase in the relative weight of the breast meat, possibly because of increased testosterone levels. By contrast, [22] demonstrated that testosterone had no direct effect on muscle mass in sparrows.

Although the feed intakes fluctuated in both groups, there were no between-group differences in average daily feed intake during the entire experimental period. Therefore, the feed intake of mule ducks was not affected by the photoperiod substantially. Silky starlings exposed to short days had higher feed intakes than those exposed to long days [23]. The feed intake in mature Chinese geese subjected to nocturnal 15-min lighting periods at 2-h intervals was similar to that in control geese subjected to 12 h of light and dark per day (12L/12D) [24]. In broilers, feed intake increased with the photoperiod during the first 21 days but was not affected later by photoperiods longer than 6 h [25]. The effect of the photoperiod on feed intake may be dependent on the species, age, or length of the day.

In Germany, migratory garden warblers (*Sylvia borin*) were exposed to 12L/12D from September to May and kept in outdoor aviaries from June to August; their basal plasma levels of triacylglycerols and glucose exhibited seasonal fluctuations, with the highest levels being between September and January and the lowest levels being between February and April [26]. The current study demonstrates that the nocturnal lighting lowered the fasting plasma glucose in mule ducks. This may be partially attributed to the high plasma testosterone levels, as well as direct effects of the photoperiod. Testosterone replacement therapy could reduce fasting glucose and glycated hemoglobin levels in patients with type 2 diabetes mellitus with hypogonadism [27]. The effect of androgen on the plasma triacylglycerol levels is not conclusive. A review suggested that androgen administration either increased or had no effect on plasma triacylglycerol levels in aging men with hypogonadism [28]. Our study demonstrates that the nocturnal light pulse did not affect the fasting plasma triacylglycerol levels in mule ducks. Uric acid is the main nitrogenous waste in birds, and plasma uric acid levels reflect the state of protein catabolism. The plasma level of uric acid was not influenced by the photoperiodic regimen in this study. Regardless of the mechanism, nocturnal 1-h lighting reduced fasting plasma glucose levels but did not affect the fasting plasma triacylglycerol and uric acid levels.

Most birds are long-day breeders: they enter the breeding season when the length of the day increases, where the signal of the long photoperiod is conveyed to hypothalamus to stimulate the secretion of GnRH, and hence to stimulate the pituitary to secrete gonadotropins and consequently to enhance the gonadal functions. Our study demonstrated that the weight of the testes and plasma testosterone levels were significantly higher in Group L+ than in Group C, and histological examination revealed that spermatogenesis had begun in Group L+ but not in Group C. The red bill, a sexual characteristic of Muscovy drakes, started to appear in the male mule ducks exposed to the nocturnal lighting. Mounting behavior was also actively exhibited by the ducks exposed to the 1-h nocturnal lighting. Our results indicate that the nocturnal lighting substantially enhanced gonadal development and function in our male mule ducks. Moreover, the length of the day (civil dawn to civil dusk) at the beginning of (12.6 h) or early in the experiment period (12.6 and 11.8 h) is short enough to reset or induce photosensitivity in mule ducks. Chickens exposed to photoperiods increasing from 6L/18D to 23L/1D demonstrated larger testes and higher plasma testosterone levels at 8 weeks than those under the constant photoperiod (23L/1D) [29]. In addition, a long-day response is believed to result from light extending into the photoinducible phase of the circadian photoperiodic rhythm (CPR), and the CPR is entrained by dawn. If the light is separated into two parts, the beginning of the long light pulse entrains the CPR and the short light pulse determines the physiological response. When the short light pulse falls in the photoinducible phase, the photoperiod is read as a “long day”; when it does not extend into the photoinducible phase, it is read as a “short day” [30]. In the current study, the supplemented lighting began from 14.2 to 14.5 h after dawn and led to a long photoperiodic effect. Therefore, the supplemented lighting is speculated to have extended into the photoinducible phase of mule ducks in this study. Our histological examination also found that the seminiferous tubule diameter was larger in Group L+ than in Group C. The histological examination also revealed that spermatogenesis did not begin in Group C ducks, but ducks in Group L+ had reached the meiosis stage in spermatogenesis. These results demonstrate that a 1-h lighting supplemented at 20:00 caused long-day effects (enhancing gonadal development and function) in mule ducks, and this regimen could be called skeleton long photoperiods.

In Group L+, the testis sections of mule ducks were observed and some small cells with a deeply stained nucleus as well as some binucleated and multinucleated cells in the central part of the seminiferous tubule were found. Snapir et al. reported that the sections of mule duck testes demonstrate organized seminiferous tubules with secondary spermatocytes as well as spermatogonia and primary spermatocytes [13]. The authors also mentioned that spermatids were rarely noted, with some having several nuclei in a single cell. This finding is similar to our current observation. However, [31] suggested that the meiosis stage in spermatogenesis in the primary spermatocytes of mule ducks enters pachytene but fails to progress beyond diakinesis-metaphase I. Nevertheless, this and previous studies were consistently unable to observe any mature spermatozoa in the mule duck testes. Therefore, the 1-h lighting supplemented at 20:00 promoted spermatogenesis in male mule ducks.

## 5. Conclusions

A 1-h lighting over 20:00–21:00, in addition to the natural photoperiod, caused long-day effects in mule ducks. In brief, it enhanced gonadal development and function and increased the relative weight of the breast meat but reduced the body weight and relative weight of the digestive tract and abdominal fat.

## Figures and Tables

**Table 1:** Effects of the nocturnal 1-h lighting on the body weight, neck circumference, and feed intake in male mule ducks.

Items	Group C ^#^	Group L+ ^#^	SEM	*p*-Value
Body Weight (g)				
15 weeks of age	3523.8	3523.8	14.4	1.000
21 weeks of age	3404.4	3205.0	57.0	0.074
Weight Loss	119.4	318.8	63.5	0.121
Daily Feed Intake (g)	143.2	141.3	3.3	0.784
Neck Circumference (cm)				
18 weeks of age	10.88	10.97	0.101	0.649
21 weeks of age	11.03	11.31	0.076	0.065

^#^ Group C was subjected to natural photoperiod only, and Group L+ was supplemented with 1-h artificial light from 20:00 to 21:00, in addition to the natural photoperiod.

**Table 2:** Effects of the nocturnal 1-h lighting on the organ weights of male mule ducks.

Items	Group C ^#^	Group L+ ^#^	SEM	*p*-Value
Live body weight (g)	3415.0	3175.0	52.186	0.015
Liver (g)	34.0	33.4	0.776	0.707
Heart (g)	26.2	28.5	1.044	0.280
Digestive tract (g)	177.0	151.4	4.526	0.001
Abdominal fat (g)	23.0	6.4	2.741	<0.001
Breast meat (g)	501.0	528.3	7.939	0.086
Testis (g)	15.7	61.0	6.338	<0.001
Relative liver weight (%)	1.00	1.03	0.023	0.522
Relative heart weight (%)	0.76	0.88	0.030	0.024
Relative digestive tract weight (%)	5.18	4.77	0.096	0.026
Relative abdominal fat weight (%)	0.67	0.20	0.077	<0.001
Relative breast meat weight (%)	14.8	16.3	0.326	0.001
Relative testis weight (%)	0.47	1.88	0.196	<0.001

^#^ Group C was subjected to natural photoperiod only, and Group L+ was supplemented with 1-h artificial light from 20:00 to 21:00, in addition to the natural photoperiod.

**Table 3:** Effects of the nocturnal 1-h lighting on the breast meat composition of male mule ducks.

Items (%)	Group C ^#^	Group L+ ^#^	SEM	*p*-Value
Moisture	74.09	74.68	0.460	0.460
Crude Protein	21.94	21.25	0.306	0.183
Crude Fat	3.78	2.37	0.351	0.142

^#^ Group C was subjected to natural photoperiod only, and Group L+ was supplemented with 1-h artificial light from 20:00 to 21:00, in addition to the natural photoperiod.

**Table 4:** Effects of the nocturnal 1-h lighting on the metabolite and testosterone levels of male mule ducks.

Items	Group C ^#^	Group L+ ^#^	SEM	*p*-Value
Glucose (mg/dL)	211.1	180.3	0.317	0.018
Triacylglycerols (mg/dL)	49.9	55.9	0.037	0.173
Uric acid (mg/dL)	3.68	4.69	0.185	0.127
Testosterone (ng/mL)	1.14	2.51	0.351	<0.001

^#^ Group C was subjected to natural photoperiod only, and Group L+ was supplemented with 1-h artificial light from 20:00 to 21:00, in addition to the natural photoperiod.

## Data Availability

All data of this study are included in the manuscript.
